# VeLeSpa: An inflected verbal lexicon of Peninsular Spanish and a quantitative analysis of paradigmatic predictability

**DOI:** 10.1007/s10579-024-09776-2

**Published:** 2024-10-09

**Authors:** Borja Herce

**Affiliations:** University of Zurich, Zurich, Switzerland

**Keywords:** Spanish, Paradigm, PCFP, Verb, Morphology

## Abstract

This paper presents VeLeSpa, a verbal lexicon of Peninsular Spanish, which contains the full paradigms (all 63 cells) in phonological form of 6553 verbs, along with their corresponding frequencies. In this paper, the process and decisions involved in the building of the resource are presented. In addition, based on the most frequent 3000 + verbs, a quantitative analysis is conducted of morphological predictability in Spanish verbal inflection. The results and their drivers are discussed, as well as observed differences with other Romance languages and Latin.

## Introduction

The last decades have witnessed a resurgence of research into purely paradigmatic relations (Aronoff, 1994; Maiden, 1992), for example in the Word-and-Paradigm tradition (Blevins, 2016; Matthews, 1965). Over the last decade, in addition, it has become easier to analyze these relations (i.e. paradigmatic word-to-word predictability) quantitatively and automatically over whole inflectional systems, thanks to advances in computing power and the development of tools (e.g. The Principal Parts Analyzer (Stump & Finkel, 2013), Qumin (Beniamine, 2018)) and resources (e.g. inflected lexicons like Unimorph [Kirov et al., 2018]) specific to our field. Much progress still needs to happen, however, regarding language documentation of highly-inflected underresearched families and languages (see e.g. Cruz et al., 2020; Herce, 2024) and, even in some major languages, regarding the development of well-curated and comparable datasets, preferably in phonological rather than orthographic form.

Indo-European and the Romance language family in particular, are generally very well studied and have been explored in an extensive qualitative philological-comparative literature (see e.g. Maiden, 2018). Regarding inflected lexicons in phonological rather than orthographic form, we have both family-wide but low-resolution^1^ ones (see ODRVM, Maiden et al., 2010), as well as high-resolution ones for some of the major languages (namely French (Bonami et al., 2014), Latin (Pellegrini & Passarotti, 2018), Italian (Pellegrini & Cignarella, 2020), Portuguese (Beniamine et al., 2021), and Romanian (Herce & Pricop, 2024)). Maybe surprisingly, however, no comparable resource exists for Spanish, and hence also no analogous quantitative analysis of morphological predictability for Spanish verbs. It is the goal of this paper to fill this gap. Section 2 explains how the Verbal Lexicon of Spanish (VeLeSpa) was built from existing ones, outlines the main challenges and decisions involved, and presents an overview of the final resource. Relying on it and on the Qumin toolkit (Beniamine, 2018), Sect. 3 presents a quantitative analysis of paradigmatic predictability in Spanish verbs. Section 4 concludes the paper and presents possible avenues for future research.

## Building VeLeSpa

The construction of a verbal inflected lexicon of Spanish started from the Spanish verbal paradigms in Wiktionary, as collected in Unimorph (Kirov et al., 2018, https://github.com/unimorph/spa/blob/master/README.md, downloaded 13/03/2023). This contains the full paradigm of 6812 verb lemmas in orthographic form. Although Spanish orthography is very transparent compared to languages like French and English, it has properties that might distort morphological alternations and predictability relations between word forms. Pairs like *re****z****o* /ˈɾeθo/ 'pray.PRS.IND.1SG' and *re****c****e* /ˈɾeθe/ 'pray.PRS.SBJV.1SG', ***qu****epo* /ˈkepo/ and ***c****abes* /ˈkabes/, ***h****uelo* /ˈwelo/ and **∅***olemos* /oˈlemos/, *and****a****ba* /anˈdaba/ and *and****á****bamos* /anˈdabamos/, etc. can give the impression of stem or stress alternations of some sort. However, the highlighted orthographic differences do not reflect any contrast in pronunciation. Conversely, orthography can gloss over differences in stress (e.g. *amo* /ˈamo/ vs *amamos* /aˈmamos/), and lead to incorrect segment alignments (e.g. *cre****zc****o* /kɾeθko/ vs *cre****c****es* /kɾeθes/). Phonological forms, and not orthographic words, are the only evidence available to native language users as they acquire the inflectional system of a language. Because of this, phonological forms are generally preferred in linguistic research.

Unlike in other Romance languages, and despite the orthographic inadequacies outlined in the paragraph above, there is largely^2^ no uncertainty in Spanish as to how a written word should be pronounced (with uncertainty only in the opposite direction, i.e. on how a given pronunciation should be encoded orthographically). Because of this, it was comparatively straightforward to transform orthographic forms into phonological forms through regular expression changes.^3^ The accentuation rules of Spanish also allow one to recover the location of word stress and (standard/prescriptive) syllabification (see Española & RAE, 2010). It must be mentioned in this respect that, although some variation exists between different varieties and even idiolects regarding hiatus vs diphthong pronunciations in many environments (e.g. *enviando* 'send.GER' /em.ˈbjan.do/ vs /em.bi.ˈan.do/, *construimos* 'build.1PL.PRS.IND' /kons.ˈtɾwi.mos/ vs /kons.tɾu.ˈi.mos/), the syllabification prescribed by Real Academia Española was adopted. This involves the former pronunciation, i.e. a diphthong, in the previous cases.

Besides the transformation into phonological transcription, further processing of the Unimorph data involved discarding multiword inflectional forms (i.e. periphrases) and those involving postposed object clitics (e.g. *da-me-lo* give.IMP-me-it', *llevemos-le* 'take.SBJV.IND.1PL- him', *diciéndo-se-lo* 'telling-him-it'), which are orthographically written as a single word, but involve syntactically independent and morphologically invariable elements (cf. *me lo da* 'me it gives'). A final adaptation involved eliminating those cases of multiple forms with the same meaning. Most of these involve form doublets one of which is not used in Peninsular Spanish (e.g. voseo 2SG forms like *llevás, corrés* besides *llevas, corres*). A small number of these involve overabundance, i.e. forms in more-or-less free variation (see Thornton 2012) like *roigo roo royo* (all prescriptively acceptable as the 1SG.PRS.IND form of 'gnaw'). In these cases a single one of these synonymous forms was kept to avoid introducing spurious complexity resulting from dialectal or idiolectal variation. Cases of defectiveness (Nevins et al., 2014) were also glossed over, as these are often gradient, and their analysis within the overall system of morphological oppositions is complicated. Focus on a single dialect is required and implemented, of course, also with regard to the phonological transcription of words. Northern Peninsular Spanish was chosen as the target variety.^4^ Because of this, /θ/ and /ʎ/ have been used rather than /s/ and /j/, avoiding also features of colloquial pronunciation like /gw/ in words starting with /w/ (e.g. *huele* 'smells'), vowel reductions, etc. Unadapted borrowings containing foreign phonemes (e.g. /ʃ/) and clusters (see Footnote 2) were also discarded.

**Fig. 1:**
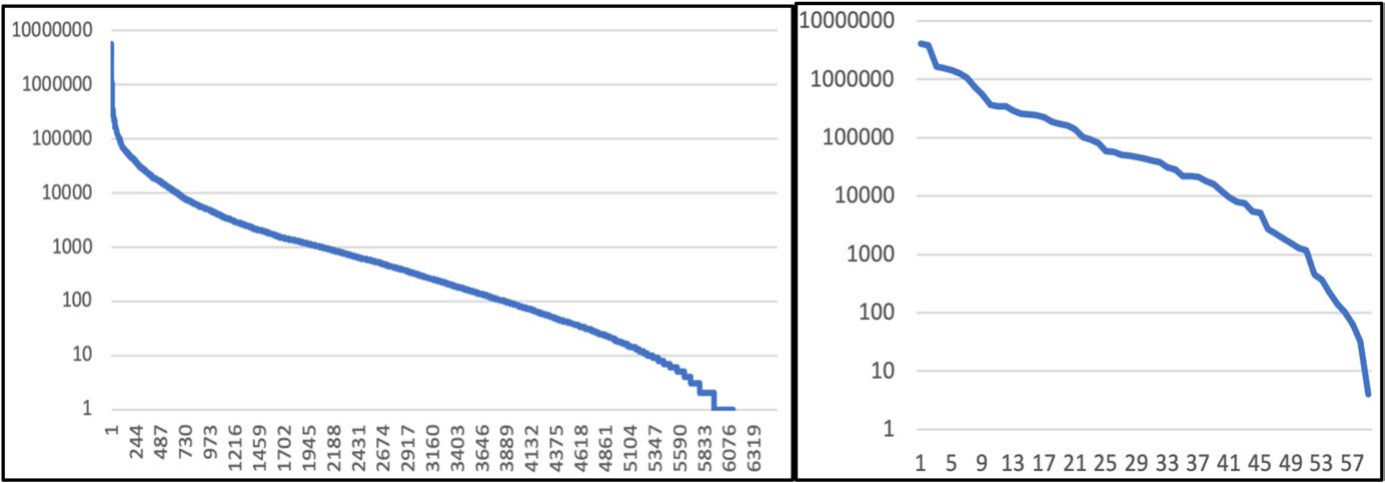
Rank frequency of the 6554 verbs in the VeLeSpa (left) and of its cells (right)

After the phonemization process and rest of decisions described, the inflected lexicon contains 6553 verbal paradigms with 63 cells each, for a total of 412,839 inflected word forms. Table 1 shows the size of VeLeSpa compared to analogous resources for verbal inflection in other Romance languages. It is evidently the case that the frequency of words in such a large lexicon is highly variable, with many of them extremely infrequent (Zipf, 1932). This (i.e. token frequency) is a factor that is crucial to the Paradigm Cell Filling Problem (Ackerman & Malouf, 2013) and beyond, and hence very important for morphological and psycholinguistic research. For this reason, the token frequency of each lemma was also supplied along its inflected forms. The frequencies of different lemmas in the Spanish corpus Corpes XXI (Version 0.93, containing 381 million words) were added, which pattern as illustrated in Fig. 1: only 8 verbs exceed 1 million tokens, 82 exceed 100,000 tokens, 650 have ≥ 10,000 tokens, 2061 have ≥ 1000 tokens, 3880 have ≥ 100 tokens, 5326 have ≥ 10 tokens, 6115 are attested at least once, and 437 verbs are completely unattested in Corpes XXI. The token frequency of cells (based also on Corpes XXI, on its Spain subcorpus) is also given in Fig. 1. This varies between 4,012,564 for the 3SG.PRS.IND and 4 for the 1PL.FUT.SBJV (note that this tense is essentially extinct in the spoken language).

**Table 1:** Size of VeLeSpa and comparable inflected lexicons in other Romance varieties

Language	Reference	Lemmas	Word forms
Latin	Pellegrini and Passarotti (2018)	3348	850,392
French	Bonami et al. (2014)	4991	253,174
Italian	Pellegrini and Cignarella (2020)	2053	108,809
Portuguese	Beniamine et al. (2021)	4987	324,214
Romanian	Herce and Pricop (2024)	7297	284,583
Spanish	VeLeSpa, this paper	6554	412,839

## A quantitative analysis of the PCFP in Spanish verbal inflection

### Data processing

With the goal of making the present analysis more comparable to the extant ones in related languages, and to shorten computation time, only the 3880 verbs with token frequency ≥ 100 were selected to perform the computations that are reported in this section. This number, necessarily arbitrary, is close to the average number of verbs analyzed in the literature (see Table 1). It corresponds to a frequency ≥ 0.263 per million words in Corpes XXI. Although the average size of the adult verbal lexicon is likely to be somewhat larger than this number (see Aitchinson, 2012; Segbers & Schroeder, 2017), this frequency threshold is justified by the fact that most native speakers do not know or use most of the verbs in the lexicon below this frequency, which hence are generally not an active part of the inflectional system learned by most speakers of Peninsular Spanish.

The free software toolkit Qumin (Quantitative Modelling of Inflection, Beniamine, 2018) was used to perform the computations. The algorithm (Python scripts) works by automatically extracting the alternations between all the different word forms of all lemmas. Within the 63-cell paradigm of Spanish, there are 3906 (= 63*62) pairs of word forms to examine for each verb. The automatic extraction of these alternations (of course comparable manual work would be impossible) allows us to find out which verbs behave the same and which behave differently, and how many classes exist, in every pair of cells in the paradigm.^5^

As the forms in Table 2 show, the INDPRS2SG and the IMP2SG in Spanish can differ in multiple ways. The morphological contrasts between /tjénes/ and /tén/, /bás/ and /bé/, or /dás/ and /dá/ are all different. The difference between /dás/ and /dá/, and /bés/ and /bé/, by contrast, is identical, with the INDPRS2SG suffixing /s/ to the IMP2SG form. This means that, whereas *tener*, *ir* and *dar* belong to different classes, *dar* and *ver* belong, for the purposes of this cell pair, to the same class.^6^

**Table 2:** Extracted morphological alternations between two cells in six verbs

lemma	gloss	‘INDPRS2SG'	‘IMP2SG'	(‘INDPRS2SG', ‘IMP2SG')
*tener*	‘have'	tjénes	tén	j_es ⇌ _
*ir*	‘go'	bás	bé	ás ⇌ é
*hacer*	‘do'	áθes	áθ	es ⇌
*decir*	‘say'	díθes	dí	θes ⇌
*dar*	‘give'	dás	dá	s ⇌
*ver*	‘see'	bés	bé	s ⇌

Once these patterns are extracted (a computationally demanding process which can take several hours), the patterns themselves can be used for quality control of the resource (checking whether exceptional of infrequent alternations are *bona fide* irregularities or mistakes in the original lexicon or its phonemization) and to perform further calculations with other Qumin scripts (e.g. conditional entropies on the basis of a single or multiple predictor cells, identifying micro and macro-classes, etc.).

### Results

An analysis of paradigmatic predictability is often presented first in terms of categorical interpredictability. Inflected word forms can be either not mutually predictable from each other (when there are multiple ways, as in Table 2, in which one form could be altered to generate the other) or they might be mutually predictable. The latter is the case, for example, with the COND3SG and the INDFUT1PL in Spanish, where ía ⇌ émos describes the morphological relation between the two cells in every single Spanish verb. Out of 3906 pairs of predictor-predicted cells in the Spanish verb paradigm, 553 (14.16%) involve no uncertainty (i.e. conditional entropy = 0). The majority of these are found to be bidirectional, i.e. conditional entropy of A given B is 0 and conditional entropy of B given A is also 0. This means that some paradigm cells can be classified into larger areas of mutual interpredictability, as is well known generally from previous literature on paradigmatic predictability (see the notion of 'stem space' in Montermini and Bonami (2013) or distillations in Stump and Finkel (2013)).

**Fig. 2:**
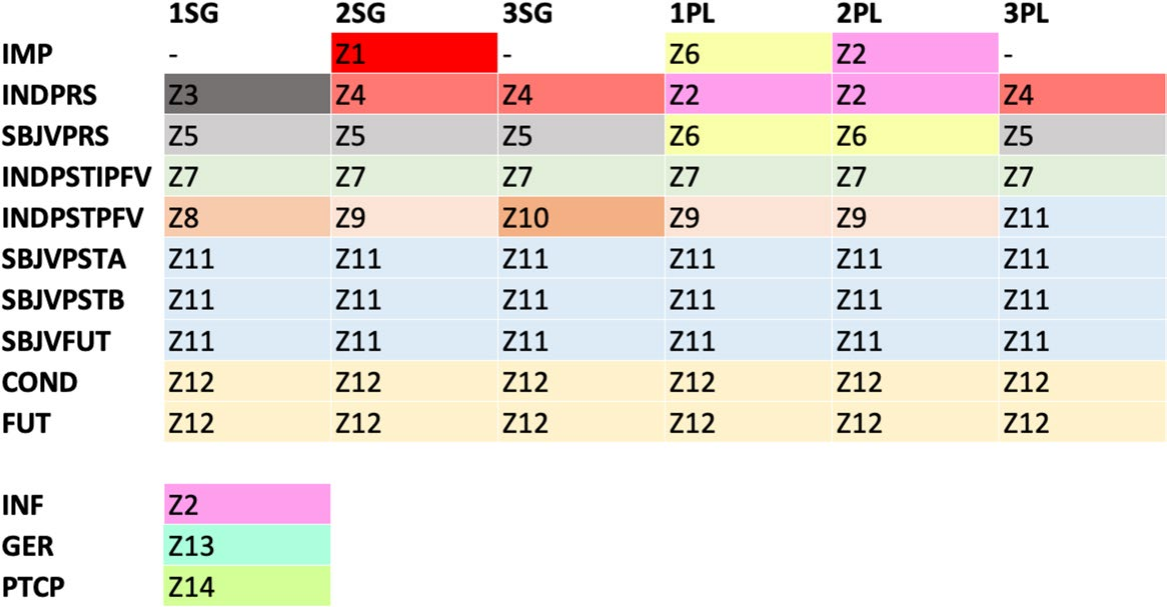
Areas of morphological interpredictability in Spanish verbal inflection

In our presently-used dataset of the highest frequency 3880 verbs in VeLeSpa, results show the 14 areas identified in Fig. 2. The use of different methodologies and datasets makes comparison difficult with previous analyses of Spanish verbal morphology (this literature has also focused largely on stems, see Boyé & Cabredo Hofherr, 2006; Palancar, 2019). Comparison with parallel research in other Romance languages, however, is more informative. The number of interpredictability areas I found in Spanish verbs, 14, is close to the number of areas in Latin (15) and in other Romance languages (15 in Italian, 14 in French and Romanian, 12 in Portuguese). Many of the patterns observed in Spanish are also observed in other Romance languages and are known to readers familiar with the literature on Romance morphomes (see e.g. Maiden, 2018). The affinity of future and conditional tenses (Zone 12 in Fig. 2), for example, is well known and diachronically unsurprising, since the tenses share an origin in verbal periphrases involving the infinitive and a tensed form of the verb 'have'. Also well-known is the morphological affinity of the future subjunctive (e.g. *amare*) and the past subjunctive (which in Spanish comes in two synonymous versions (e.g. *amara* (A) and *amase* (B) 'love'). This affinity derives from the Latin perfectum tenses, all of which shared the same stem. All person-number forms within the imperfect indicative (e.g. *amabas* and *amábamos*) are also mutually predictable in Spanish, as in other Romance languages like Italian and Portuguese, and in their ancestor Latin.

More broadly, Spanish is also like other Romance languages in generally not showing natural-class, but instead morphomic (see Aronoff, 1994; Maiden, 2018; Herce, 2023) distributions of its morphological domains. Focusing on multi-cell areas (the ones where naturalness or unnaturalness can be distinguished), all of them except the indicative past imperfect (Z7) can be taken to be unnatural: 2PL.IMP + 1PL.PRS.IND + 2PL.PRS.IND (Z2), 2SG.PRS.IND + 3SG.PRS.IND + 3PL.PRS.IND (Z4), SG.PRS.SBJV + 3PL.PRS.SBJV (Z5), etc. The patterns of interpredictability also vary strongly from one tense to the other (more on this below), which suggests that these domains are morphosyntactically largely arbitrary, and might be regarded as an inherited purely morphological property.

**Fig. 3:**
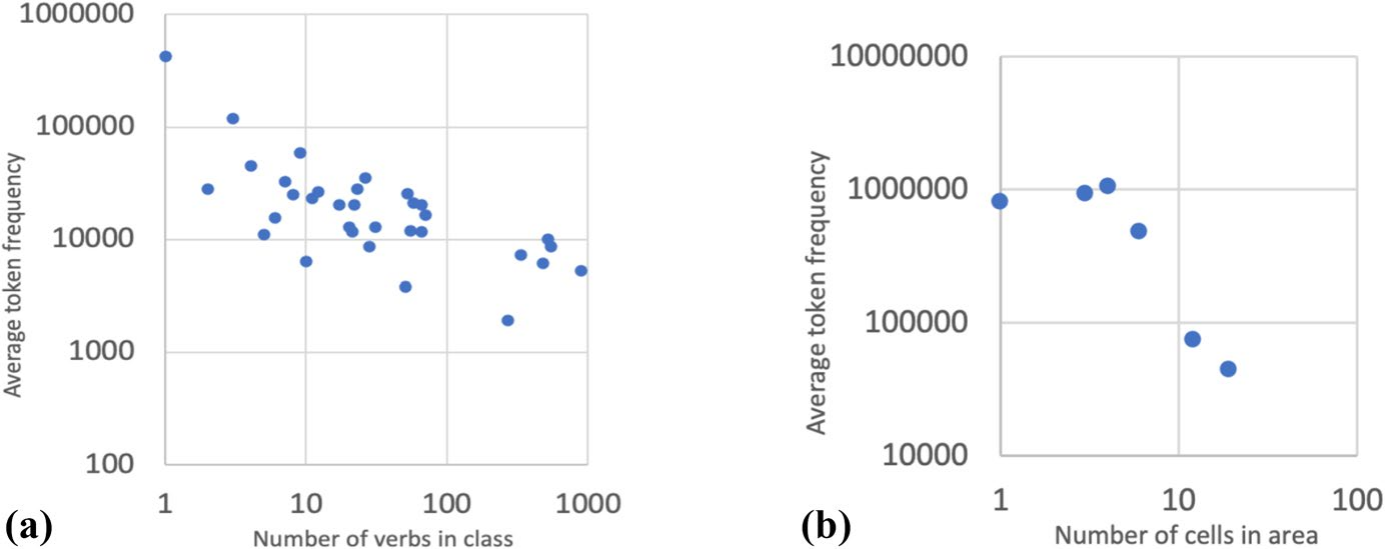
**a** Regularity and frequency in lemmas. **b** Regularity and frequency in cells

More broadly still, Spanish is seen to behave not only like other Romance languages but also in line with well-known cross-linguistic tendencies in that a strong correlation is observed between frequency and irregularity (Herce, 2019; Wu et al., 2019). This is the case of both inflection classes and paradigm cells. Regarding the former (see Fig. 3a), verbs from singleton or low type frequency classes are more frequent on average than verbs from large classes. Regarding the latter (see Fig. 3b), cells from single-cell (Z1, Z3, Z8, Z10, Z13, Z14 in Fig. 2) or small predictability areas (Z4, Z6, Z9, Z2, Z5) are more frequent on average than cells from larger morphological domains (Z7, Z12, Z11). It is a logical necessity that, given the Zipfian nature of linguistic input (Blevins et al., 2017), infrequent verbs and forms are less likely to be learned by rote and more likely to be produced online following more general rules of the language, thus losing irregular morphological traits (see Lieberman et al., 2007). Spanish verbal inflection is not, and arguably cannot be, an exception to this (Herce, 2016).

In this sea of similarity, the main differences between the predictability patterns from Spanish and those of its closest siblings concern the present indicative and the past indicative. The former is, in every other Romance language and Latin, the tense which is split into most areas of interpredictability (6 in Italian, 5 in Portuguese and Latin, 4 in French). This is not so in Spanish, where only 3 domains are found: 1SG, 2SG/3SG/3PL, and 1PL/2PL. The tense is hence simpler, in this respect, than in the rest of Romance. It is striking, or at least unparalleled in the family, that such frequent cells as 2SG, 3SG, and 3PL present indicative are all mutually predictable. The past indicative shows the opposite tendency. Whereas other Romance languages split this tense in 2 domains of interpredictability at most, Spanish boasts 4 distinct domains here, more than in the vastly more frequent present. This membership of past indicative cells into different interpredictability domains is caused, among others, by the alternations displayed in Table 3. As can be seen, there are multiple cross-classifying ways in which cells from those domains can differ from each other in different verbs.

**Table 3:** Some past indicative inflected forms and alternations in Spanish

INDPST1SG	INDPST2SG	INDPST3SG	INDPST3PL	'INDPST1SG', 'INDPST2SG'	'INDPST1SG', 'INDPST3SG'	'INDPST1SG', 'INDPST3PL'
fwí	fwíste	fwé	fwéɾon	⇌ ste	í ⇌ é	í ⇌ éɾon
estúbe	estubíste	estúbo	estubjéɾon	ú_ ⇌ u_íst	e ⇌ o	ú_e ⇌ u_jéɾon
túbe	tubíste	túbo	tubjéɾon	ú_ ⇌ u_íst	e ⇌ o	ú_e ⇌ u_jéɾon
púde	pudíste	púdo	pudjéɾon	ú_ ⇌ u_íst	e ⇌ o	ú_e ⇌ u_jéɾon
úbe	ubíste	úbo	ubjéɾon	ú_ ⇌ u_íst	e ⇌ o	ú_e ⇌ u_jéɾon
díxe	dixíste	díxo	dixéɾon	í_ ⇌ i_íst	e ⇌ o	í_e ⇌ i_éɾon
dí	díste	djó	djéɾon	⇌ ste	í ⇌ jó	í ⇌ jéɾon
bí	bíste	bjó	bjéɾon	⇌ ste	í ⇌ jó	í ⇌ jéɾon

Apart from discussing the areas of perfect interpredictability, we can also present and comment on the overall uncertainty (i.e. conditional entropies) involved in predicting between word forms among these domains. Table 4 shows how difficult it is to predict the form indicated by the column on the basis of the form indicated by the row. Thus, for example, the difficulty of predicting Z2 on the basis of Z1 is 0.066 bits. Some interesting observations on the results from Table 4 are the following: Zone 3, i.e. the 1SG.PRS.IND is, as Table 4 shows, the least informative cell (see also Table 5). This is also clearly the case in Portuguese (see Beniamine et al., 2021).^7^ Also shared with the other national standard Ibero-Romance language (and beyond in this case) is the fact that the rhizotonic cells of the present (the so-called *N*-morphome, i.e. domains Z1, Z3, Z4 and Z5) are the least predictable from other cells in the paradigm (see their columns in Table 5). In Spanish, this is largely the result of unpredictable stem-vowel diphthongizations (e.g. *costar cuesta, cerrar cierra,* but *cortar corta, cenar cena*) that distinguish the rhizotonic (i.e. root-stressed) forms from the rest.

**Table 4:**
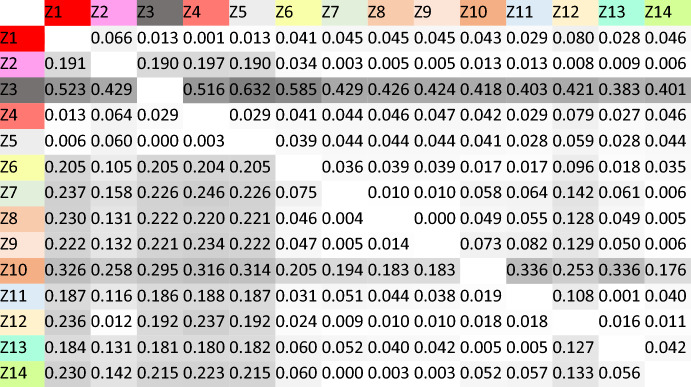
Conditional entropies (column given row) between the distillations in Fig. 2

**Table 5:**
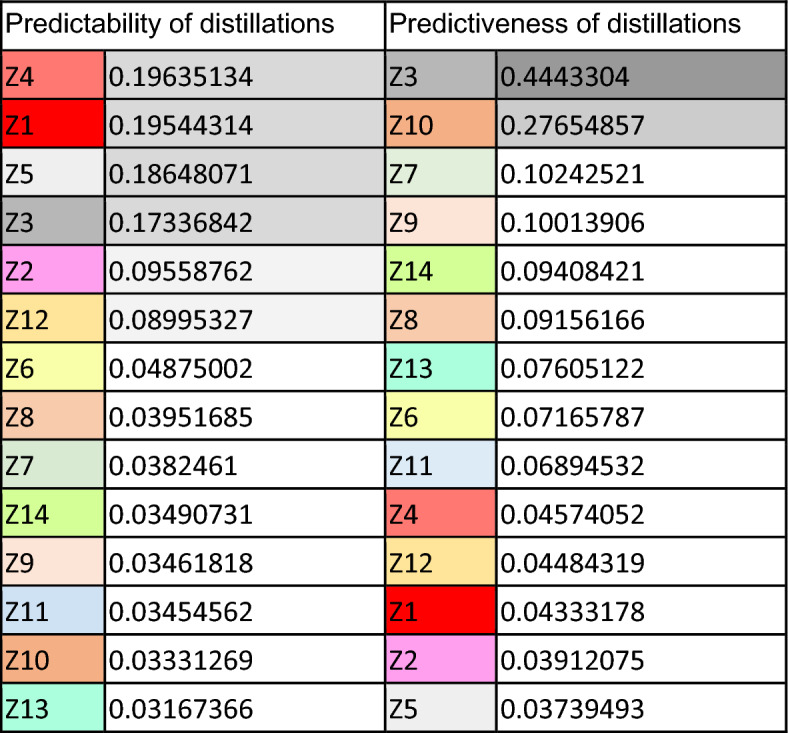
Average predictability and predictiveness of Spanish distillations

Alongside the areas of higher unpredictability (gray), Table 4 also shows those of low uncertainty (white). Some distillations that come close to perfect interpredictability are some of the past indicative domains mentioned in Table 3 (namely Z8, Z9 and Z11), as well as Z11 (e.g. *dijeron, tuvieron, hicieron, pusieron, amaron, vivieron, midieron, pudieron*) and Z13 (*diciendo, teniendo, haciendo, poniendo, amando, viviendo, midiendo, pudiendo*).

The working hypothesis embedded in the PCFP is that perfect and high predictability relations must be picked up by language users as they learn their language and must be actively used when they produce unobserved forms online on the basis of other forms. Predictability relations, hence, must drive analogical morphological changes in diachrony as well. The morphological affinity of Z11 and Z13 in Spanish, for example, must be the reason why we sometimes witness the emergence of analogical gerunds like *dijendo, *tuviendo, *hiciendo, *pusiendo, etc. in some Peninsular Spanish varieties, where these innovative forms replace standard *diciendo, teniendo, haciendo, poniendo,* etc. (see Pato & O'Neill, 2013). The new forms, which borrow the stem from the Z11 former-perfectum domain, represent an elimination of the very few unpredictable alternations between the two distillations. In the overwhelming majority of Spanish verbs, e.g. *amaron amando, corrieron corriendo, vivieron viviendo, murieron muriendo, vinieron viniendo, pidieron pidiendo*, etc. a simple ɾo_ ⇌ _do captures the change between 3PL past indicative and the gerund. Only in a few irregular high frequency verbs (e.g. *dijeron diciendo, tuvieron teniendo, hicieron haciendo, pusieron poniendo*, etc.) there are additional changes in the stem as well. Erasing these, as the mentioned nonstandard varieties do, would bring Z11 and Z13 into the same distillation, which would reduce the overall complexity of the Spanish verbal inflectional system. This (i.e. regularity) is generally taken to be the "purpose" of analogical morphological change (see Sturtevant, 1947).

Table 5 shows the predictability and predictiveness of all distillations. The former refers to the uncertainty involved in predicting a given cell from others in the paradigm (i.e. how difficult is it to guess X?). The latter refers to the uncertainty involved in predicting other forms from a given cell (i.e. how difficult is it to guess *from* X?). As in other Romance languages, it can be observed that differences in predictiveness are much more pronounced than differences in predictability. It awaits further exploration whether this is a property of Romance verbs, or a cross-linguistic trend motivated by the necessity to predict all word forms (but not so much for all word forms to be predictive). The average implicative entropy between Spanish cells is 0.073787 (0.12109 between distillations), which is somewhat lower than in the other Romance languages that have been analyzed in a comparable way: 0.28 for Latin (Pellegrini & Passarotti, 2018), 0.18 for French, 0.17 for Portuguese (Beniamine, 2018), and 0.14 for Romanian (Herce & Pricop, 2024).

It is tempting, but highly speculative at present, to link these differences to the sociolinguistic history of the different languages. There is abundant literature suggesting a link between high levels of contact or historical L2 adult language acquisition and morphological simplification (Bentz & Winter, 2013; Kusters, 2003; Lupyan & Dale, 2010a; McWhorter, 2007; Trudgill, 2011). Spanish is the most widely spoken Romance language and has historically expanded dramatically from its small home in North-Western Castille to close to 500 million people nowadays, spread across 22 countries covering close to 10% of the global land area. This might provide a partial explanation to the greater degree of simplification found in Spanish. Of course, future research should be conducted to check that these differences are not due to properties specific to the inflected lexicons used or other factors like chance.

## Conclusion

This paper has presented a novel resource, VeLeSpa: A verbal inflected lexicon of Peninsular Spanish verbs in phonological form. The resource, built for computational use and thoroughly checked, contains 6553 verbal paradigms with 63 cells each, for a total of 412,839 inflected word forms. The full lexicon and the file with the distinctive features of Spanish phonemes is made freely available for further not-for-profit linguistic research at https://osf.io/gne73/?view_only=7fc0ddb495aa4cb8b3b50b2a4dbfdb85.

The second part of this paper, based on a high-frequency subset of the paradigms in VeLeSpa, contains a quantitative analysis of morphological predictability in Spanish verbs, using the toolkit Qumin (Beniamine, 2018). The results allow us to compare Spanish to other Romance languages for which the same analyses have been conducted, such as Latin, French, Italian, and Portuguese. In terms of the number of mutual-predictability domains in the paradigm (14), Spanish shows a degree of complexity very similar to other languages in its family. The structuring principles of paradigmatic predictability in the language are found to be largely morphomic, reflecting the accidents of history (e.g. historical sound changes), rather than morphosyntactic or semantic. This agrees with received wisdom from the philological and Romance historical-comparative literature (e.g. Maiden, 2018). At the same time, and in line with cross-linguistic findings, results also reveal a correlation between frequency and irregularity (see Fig. 3).

Alongside these similarities, some differences have also been found between verbal morphological predictability in Spanish and its sister languages. While the PRS.IND tense has been found to be simpler than in the rest of Romance, the PST.PFV.IND shows the opposite trend, i.e. more complexity, as measured by number of interpredictability domains, than the other languages in the family that have been explored so far.^8^ Across all cells, Spanish seems to be generally simpler (i.e. shows lower average conditional entropies) than other Romance languages. It remains to be seen whether this should be attributed to its rapid spread and a large number of incoming L2 speakers from the tenth century onwards.

These subtle differences notwithstanding, stability prevails regarding the paradigmatic and morphological predictability structures observed across Romance verbs. The notable stability of paradigmatic structure has long been argued for in much qualitative diachronic literature, in Romance and beyond (e.g. Meillet, 1958; Nichols 1996; Maiden, 2018). It is, thus, striking, how similar the systems of contemporary Spanish, Portuguese, French, and Italian are after nearly 2 millennia of separate evolution. Future research would profit from pursuing a 'diachronic turn' (Blevins, 2013) in the quantitative exploration of paradigmatic predictability. This is urgently needed to test whether these relations indeed bear a strong long-lasting phylogenetic signal, as most extant synchronic research suggests, and to find out whether or how they can inform language taxonomies and reconstruction, alongside, or in addition to, traditional methods based on systematic sound correspondences. This shall be left for now to future research.

## Footnotes

1 The ODRVM has full and partial paradigms from 73 Romance varieties but a very low number of verbs per variety (24.5 on average, 43 for Spanish). It also contains too much noise for its reliable computational use, for example mistakes such as *moriendo*, rather than *muriendo* as the gerund form or *morir* 'die' in Spanish, and variable phonemic transcription conventions.

2 Exceptionally, the grapheme 'x' can be pronounced as either /x/ or /ks/. Borrowed words may also preserve a spelling that does not correspond to the one in native words. In the original Unimorph dataset, for example, we can find among others *crackear**, **hackear**, **shockear**, **fotoshopear**, **twitear*, etc. This introduces another source of occasional uncertainty regarding the pronunciation of some graphemes or character sequences (e.g. 'sh' cf. *deshacer* or 'h' cf. *hacer*).

3 Being exhaustive, orthographic r was changed into phonemic ɾ, rr into r, x into ks, j into x, z into θ, y into j, v into b, ñ into ɲ, ll into ʎ, and h was deleted. Turning to context-dependent adaptations, que was changed into ke, qui into ki, ce into θe, ci into θi, ge into xe, and gi into ji. Taking syllabification into account (signalled unmistakably along with stress through Spanish accentuation), non-syllabic i was changed into j, and nonsyllabic u (also ü) into w.

4 This focus is largely derived from the fact that this variety is the author's L1 and should not be taken to be associated with any ideological or prescriptive stance. Focus on a phonologically and morphologically conservative variety like this one, however, is also useful. While phonological mergers (such as between /s/ and /θ/) and morphological innovations (such as the replacement of the original 2PL *vosotros* and its agreeing forms by *ustedes* and its associated 3PL agreeing forms) are easy to apply to a lexicon, the reintroduction of lost forms and phonemes is much more challenging.

5 For a more detailed explanation of how the alternations are identified, for example when multiple descriptions are possible (e.g. /éɾes/ /sé/: éɾe_ ⇌ _é, or _ɾes ⇌ s_) see Beniamine et al. (2021).

6 The algorithm also provides the phonological context where a given alternation occurs if a coherent one can be identified (note that, apart from the inflected lexicon itself, a different file needs to be provided to Qumin specifying the language's phonemes and their description in terms of distinctive features. This file is also provided in the supplementary material accompanying this paper: https://osf.io/gne73/?view_only=7fc0ddb495aa4cb8b3b50b2a4dbfdb85

7 Not so in Italian (Pellegrini and Cignarella 2020), where other paradigm cells have innovated so-called ‘superstable' markers (Wurzel 1978) like the 2SG.PRS.IND -*i*, and the 1PL.PRS.IND -*iamo.*

8 The morphological simplicity of the PST.PFV.IND beyond Spanish could be partially explained by the obsolescence of this tense in many Romance languages (e.g. French and Italian), where it is often replaced, as in German, by a present perfect construction involving the participle and a conjugated auxiliary verb 'be' or 'have'.
